# Comparison of a 100-pF Capacitor With a 12 906-Ω Resistor Using a Digital Impedance Bridge

**DOI:** 10.1109/tim.2021.3139709

**Published:** 2021

**Authors:** Mona Feige, Stephan Schlamminger, Andrew D. Koffman, Dean G. Jarrett, Shamith Payagala, Alireza Panna, Bryan C. Waltrip, Michael Berilla, Frank Seifert, Yicheng Wang

**Affiliations:** Quantum Measurement Division, National Institute of Standards and Technology, Gaithersburg, MD 20899 USA.

**Keywords:** ac voltage ratio, digital bridge, impedance standard, lock-in detector, noise cancellation

## Abstract

We tested a digital impedance bridge in a hybrid structure for comparison of a capacitor with a resistor where the impedance ratio was measured in two separate parts. The modulus of the impedance ratio was matched arbitrarily close to the input-to-output ratio, in magnitude, of a two-stage inductive voltage divider by adjusting the operating frequency of the bridge; the residual deviation between the two together with the phase factor of the impedance ratio was measured using a custom detection system based on a four-channel 24-bit digitizer. The ratio of the inductive voltage divider was calibrated, *in situ*, using a conventional four-arm bridge with two known capacitors. Fluctuations of the source voltages were largely removed through postprocessing of the digitized data, and the measurement results were limited by the digitizer error. We have achieved an overall bridge resolution and stability of 0.02 μF/F in 2 h for measuring a 100-pF capacitor relative to a 12 906-Ω resistor at 1233 Hz. The relative combined standard uncertainty (*k* = 1) is 0.13 μF/F, dominated by the digitizer error.

## I. INTRODUCTION

Digital techniques can be readily used to generate two synchronized ac voltages with a phase difference of *π*/2. The digital bridges, based on such ac sources, have the potential to greatly simplify comparisons between a capacitor and a resistor. Precise measurements of such impedance ratios are critical to developing quantum-based impedance standards. The present status of the digital bridges as compared with the traditional transformer-based impedance bridges has been recently reviewed [1]. The latter still provides measurements with the highest accuracy for the most demanding applications, including the realization of the capacitance unit from calculable capacitors or the ac quantized Hall resistance (QHR) through a quadrature bridge [2]–[4]. However, the digital bridges have been noticeably improving for impedance comparisons, offering many advantages through computer control and automation [5]–[11]. In particular, Josephson arbitrary waveform synthesizers establish a quantum-based voltage ratio standard that can be used for impedance comparisons at any phase angle [5], [6]. Digital signal sources custom-designed for impedance bridges have also shown great promise. A dualchannel ac voltage source with amplitude ratio stability better than 0.01 μV/V and a phase resolution of 0.2 μrad at 1 kHz has been reported [10]. A fully-digital four-terminal-pair (4TP) bridge, using such a custom-designed voltage ratio source for reference, has been reported for RC comparisons with a 1:1 magnitude ratio with a combined uncertainty of 9.2 × 10^−8^, showing great promise for the realization of the unit of capacitance from an ac QHR standard [11]. Another interesting approach [12] is to use commercial synthesizers that are then stabilized with a negative feedback loop, minimizing the bridge error signal.

**Fig. 1.**
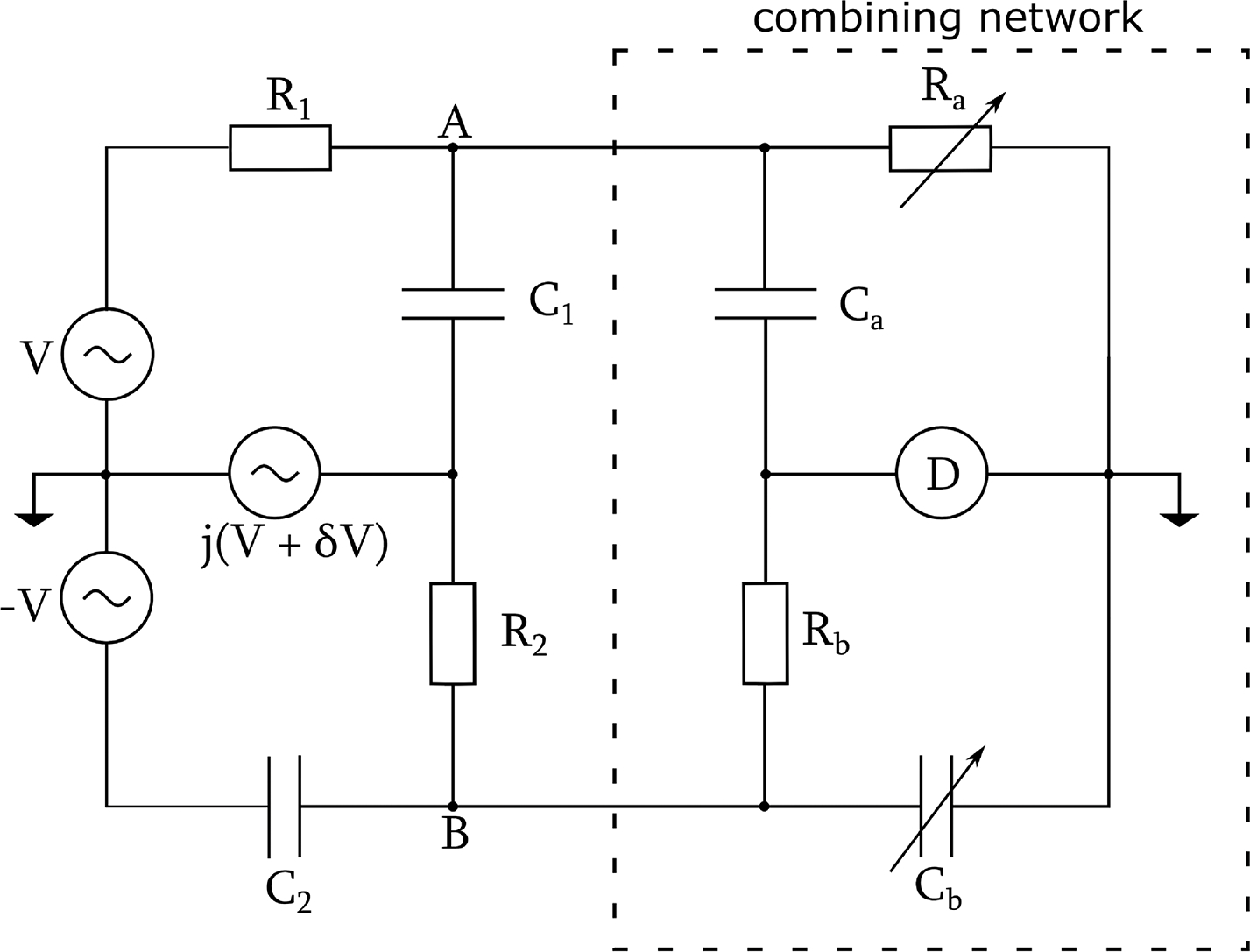
Schematic of Quad bridge with combining network.

When the voltage ratio of two synthesized sources is used directly as the reference for impedance ratio measurements, as described in the literature [5]–[11], the stability of the voltage ratio can become a major limiting factor for the overall bridge performance. It appears that an underexplored research area is to mimic in the digital domains some analog techniques that are commonly used in the analog bridges to correlate and combine detector voltages, enabling suppression of the effect of source fluctuations. Let us consider the Quad bridge [2], [3], shown in Fig. 1, as an example. The complex impedance ratio of a resistor and a capacitor, with a phase of *π*/2, cannot be measured with high accuracy with a single quadrature bridge because the required voltage ratio at a phase angle of *π*/2 cannot be accurately produced in an analog bridge. However, two such ratios in sequence, forming a double quadrature bridge with a total phase shift of *π*, can be measured with high accuracy using a transformer ratio as reference. It is important to observe that although the overall accuracy of a Quad bridge can be very high, the error voltages of the individual quadrature bridges at points A and B (see Fig. 1) fluctuate significantly due to the inevitable fluctuation of the quadrature voltage represented by δV. An elegant feature of the Quad bridge is to combine the error voltages with an RC combining network such that it forms, with the main bridge components, a twin-T network from the quadrature voltage to the detector, D; the twin-T network is a notch filter and can be adjusted so that D is immune to δV at the fundamental frequency of the bridge excitation.

The Quad bridge can be simplified using digital techniques. Specifically, if the detector voltages at points A and B (see Fig. 1) are digitized, their correlation can be analyzed in post-processing and the function of the analog combining network can be replaced by software algorithms. One can further argue that if the detector voltage of a single quadrature bridge is synchronously digitized with the source voltages, their correlation can also be analyzed to suppress the source fluctuations. This article describes our research in this direction, aiming to develop a simple digital bridge for RC comparisons.

## II. BRIDGE SETUP

**Fig. 2.**
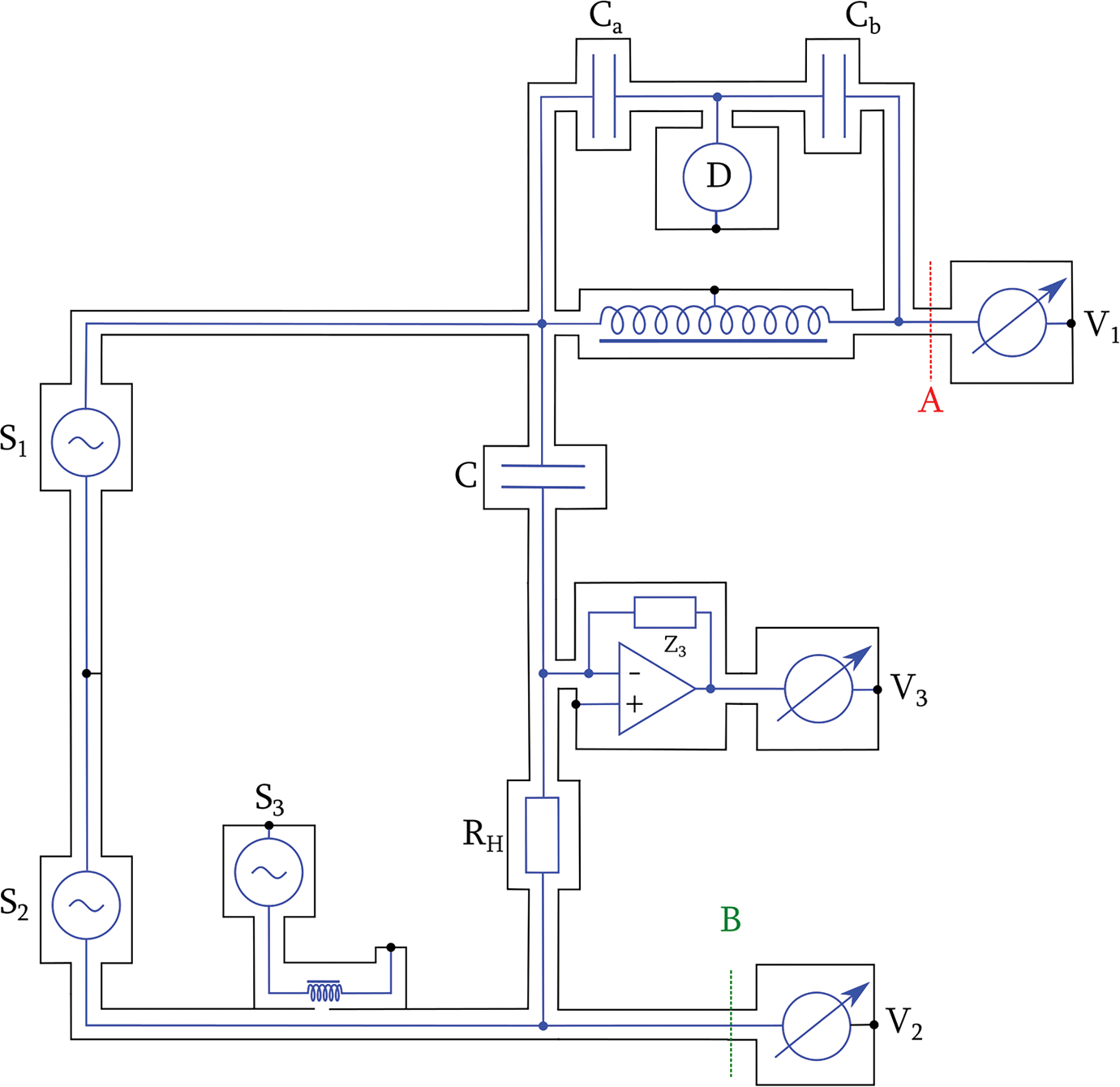
Schematic of digital impedance bridge for comparison of $Z_{1}={\left(j\omega C\right)}^{-1}$ and $Z_{2}=R_{H}$ (C=100pF and $R_{H}=12\;906\;\;\Omega$ at a frequency near 1233 Hz. $Z_{3}$ is the feedback resistor of the current amplifier. $S_{1}$ (amplitude = 10 V, phase = 90°), $S_{2}$ (amplitude = 0.1 V, phase = 0), and $S_{3}$ are waveform generators.$V_{1}$, $V_{2}$ and $V_{3}$ are ac voltmeters. $V_{1}$ (amplitude = 0.1 V, phase = −90°) and $V_{2}$ (nominally, amplitude = 0.1 V, phase = 0) are connected to the high-potential ports (A and B) and are periodically switched to minimize the effect of their gain drift. $V_{3}$ is adjusted such that $S_{3}$ is nominally 0. Coaxial chokes (omitted for clarity) are placed in every unwanted loop in the bridge circuit [13].

The digital impedance bridge, shown in Fig. 2, is designed for comparisons between a 4TP Vishay^1^ resistor, with a nominal value of $R_{H}=12\;906\;\Omega$, in an air bath at 23 °C, and a two-terminal-pair (2TP) Andeen–Hagerling capacitor, with a nominal value of C=100pF. The impedance of the capacitor and the resistor are represented with $Z_{1}=(1/j\omega C)$ and $Z_{2}=R_{H}$, respectively, and the associated impedance ratio is represented by a complex number, $re^{j\theta }=\left(Z_{1}/Z_{2}\right)$. The low port of the 2TP capacitor was connected directly to the low-current port of the resistor without a combining network by following a method described by Small *et al.* [14] to compare 4TP resistors with 2TP capacitors. A current amplifier (Femto DLPCA-200) with transimpedance of $Z_{3}$, which is used to detect the bridge error voltage, was connected to the low-potential port of the 4TP resistor. Hence, the cable and the contact resistance between the low-current port of the resistor and the low port of the 2TP capacitor were then considered part of the capacitance standard. As long as the defining planes are applied consistently in calibrations, the inclusion of contact resistance only affects the dissipation factor of the capacitor slightly, with a negligible contribution to the uncertainty of the capacitance measurements.

We used two phase-locked channels ($S_{1}$ and $S_{2}$) of a Keysight 33500B waveform generator as the main sources to excite the bridge through a 2TP current loop connecting to the high-current ports of $Z_{1}$ and $Z_{2}$, applying root mean square (rms) voltages of 7.07 V and 70.7 mV, respectively, to the capacitor and the resistor at a frequency near 1233 Hz. To overcome the limited resolutions of the generator outputs, another synchronized 33500B generator $\left(S_{3}\right)$ was used to inject a fine adjustment signal through a 10 000:1 injection transformer inserted into the lower excitation arm. An external time base was used for both generators with their 10-MHz reference signal locked to the Global Positioning System.

The modulus of the nominal impedance ratio is 100. To avoid the digitizer nonlinearity of sampling the excitation voltages with different amplitudes, a two-stage inductive voltage divider (IVD), with its input-to-output ratio, $k_{o}$, having a nominal value of −100, was added between the high port of the capacitor and the voltage measurement system. The operating frequency of the bridge was fine-tuned such that the modulus of the impedance ratio, r, was arbitrarily close to $k_{o}$ in magnitude and the sampled $V_{1}$ and $V_{2}$ were nominally equal in amplitude. The IVD ratio may slightly depend on the loading condition and therefore was calibrated, *in situ*, using a conventional four-arm bridge with two capacitors, $C_{a}$ and $C_{b}$, of nominal values of 1 and 100 pF, respectively. A small micrometer-controlled trim capacitor added in parallel to $C_{b}$ was used to null the in-phase component of the bridge error.

The IVD output (A) and the low-potential port (B) of $Z_{2}$ form a 2TP potential loop of a digital bridge with two voltage detectors through a custom coaxial switching fixture, which was described previously [15]. The two detectors were periodically interchanged to minimize the effect of their gain drift. A small loading change at A and B is equivalent to a small change of the excitation voltage ratio, which is suppressed in the digital domain by correlation with the bridge error signal.

We used a Keysight DAQM909A, a four-channel 24-bit digitizer module in a Keysight DAQ973A data acquisition system, to simultaneously sample $V_{1}$, $V_{2}$, and $V_{3}$, preserving the relative phase difference of the three signals. The digitizer was set with differential input, a sampling rate of 800 000 samples/s, and a record length of 2 400 000 samples for each measurement. The analog bandwidth of the digitizer is approximately 125 kHz. The amplitude and the phase of each sampled voltage were determined using an algorithm of three-parameter least-squares fit as described in IEEE Standard 1057–2017 [16].

The digital bridge (see Fig. 2) relies on accurate measurements of voltage ratios to determine the phase factor of the impedance ratio, $e^{j\theta }$. In the ideal case, the excitation sources would be adjusted to balance the bridge, such that for any measured voltage, $V_{2}$, at the high-potential port of $Z_{2}$, the measured voltage, $V_{1}$, which is scaled down by the IVD from the high-potential port of $Z_{1}$, would be equal to a perfect value $V_{1p}$, achieving the condition of equal current through the two impedances under comparison. The balanced equation is

(1) $$\frac{Z_{1}}{Z_{2}}=-\frac{k_{o}V_{1p}}{V_{2}}.$$

In practice, the balance is never perfect, and the source drift always exists. The combined effect can be represented by an error voltage, δV, superimposed on the ideal voltage $V_{1p}$, and we have $V_{1}=V_{1p}+\delta V$. The effect of the error voltage is automatically balanced through the feedback resistor $Z_{3}$ of the current amplifier. The common low-potential port is kept at virtual ground, and the detected error voltage, $V_{3}$, relates to δV through

(2) $$\frac{Z_{1}}{Z_{3}}=-\frac{k_{o}\delta V}{V_{3}}.$$

The phase difference between $V_{3}$ and δV is approximately 90°. We define the gain factor $g=\left(j/k_{o}\right)\left(Z_{1}/Z_{3}\right)$. Hence, $j\delta V+gV_{3}=0$. The bridge dynamics can be understood as a superposition of the two voltage-balancing actions governed by (1) and (2).

## III. TEST RESULTS AND DISCUSSIONS

### A. Equal Voltage Test

The measurement accuracy of the DAQM909A for ac voltage ratios depends on not only the resolution of the digitizer but also the gain stability of the input amplifiers. To determine the limitations of the digitizer, we connected two input channels of a DAQM909A in parallel to the same sinewave voltage, with an rms value of 0.1 V at 1 kHz, similar to the tests described in the previous article [15] when the two SR860 lock-in detectors were used to measure large ac signals. The best results were obtained when the two input channels, set at the 0.3-V input range, were periodically interchanged through the coaxial switching fixture, creating two virtually identical digitizing channels. The Allan deviation of the measured unity voltage ratio as a function of the averaging time follows a straight line in a log-log plot, with its slope consistent with averaging over white noise. It reaches below 0.01 μV/V in approximately 4 h, about a factor of 10 lower than what was achieved using the SR860s.

### B. Digitized Bridge Voltage

**Fig. 3.**
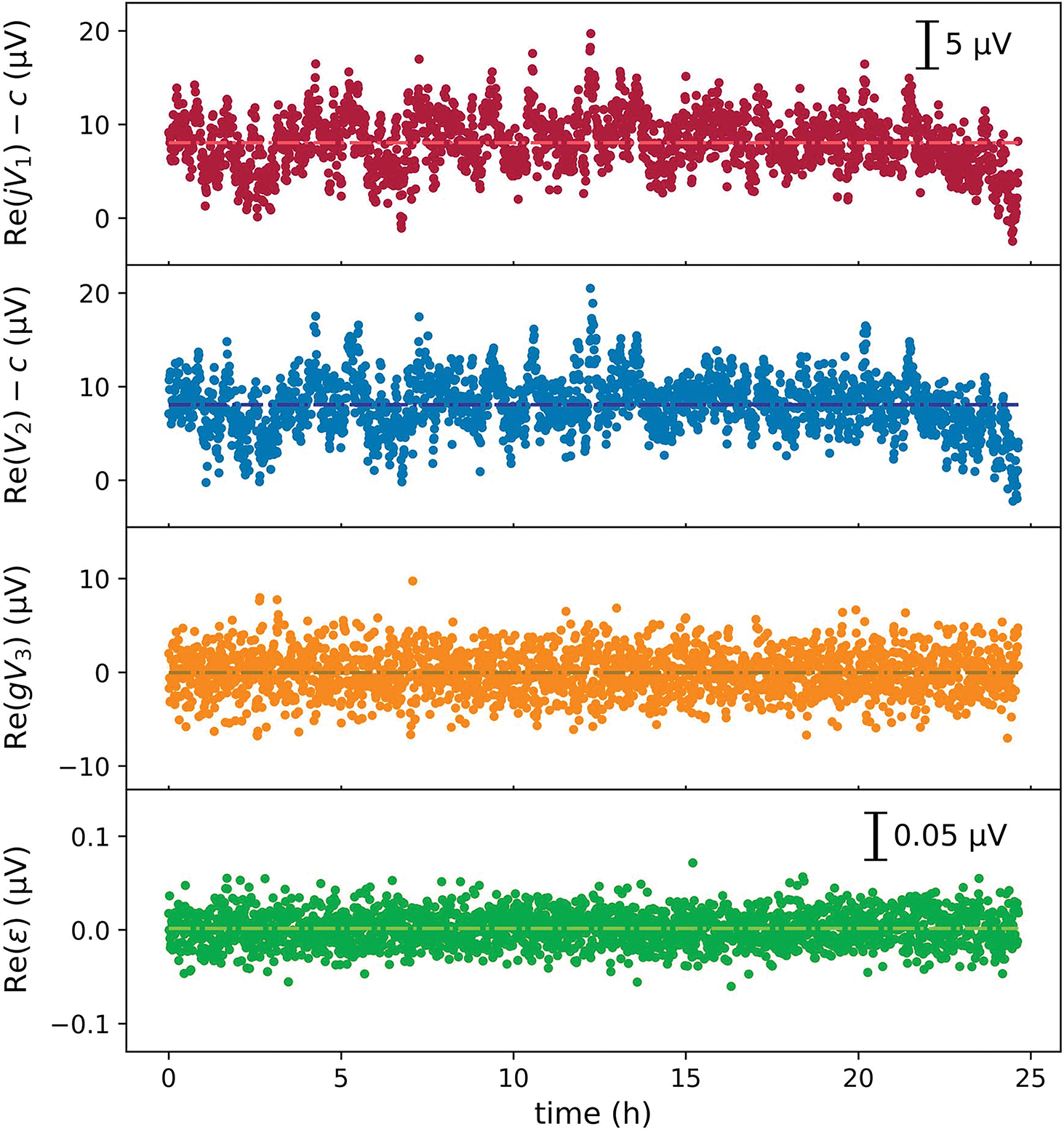
Real components of recorded voltages as a function of time: 1) $jV_{1}$ 2) $V_{2}$; 3) $V_{3}$ scaled with the gain factor; and 4) ε. $jV_{1}$ and $V_{2}$ are shifted by $c=10^{5}$ μV.

**Fig. 4.**
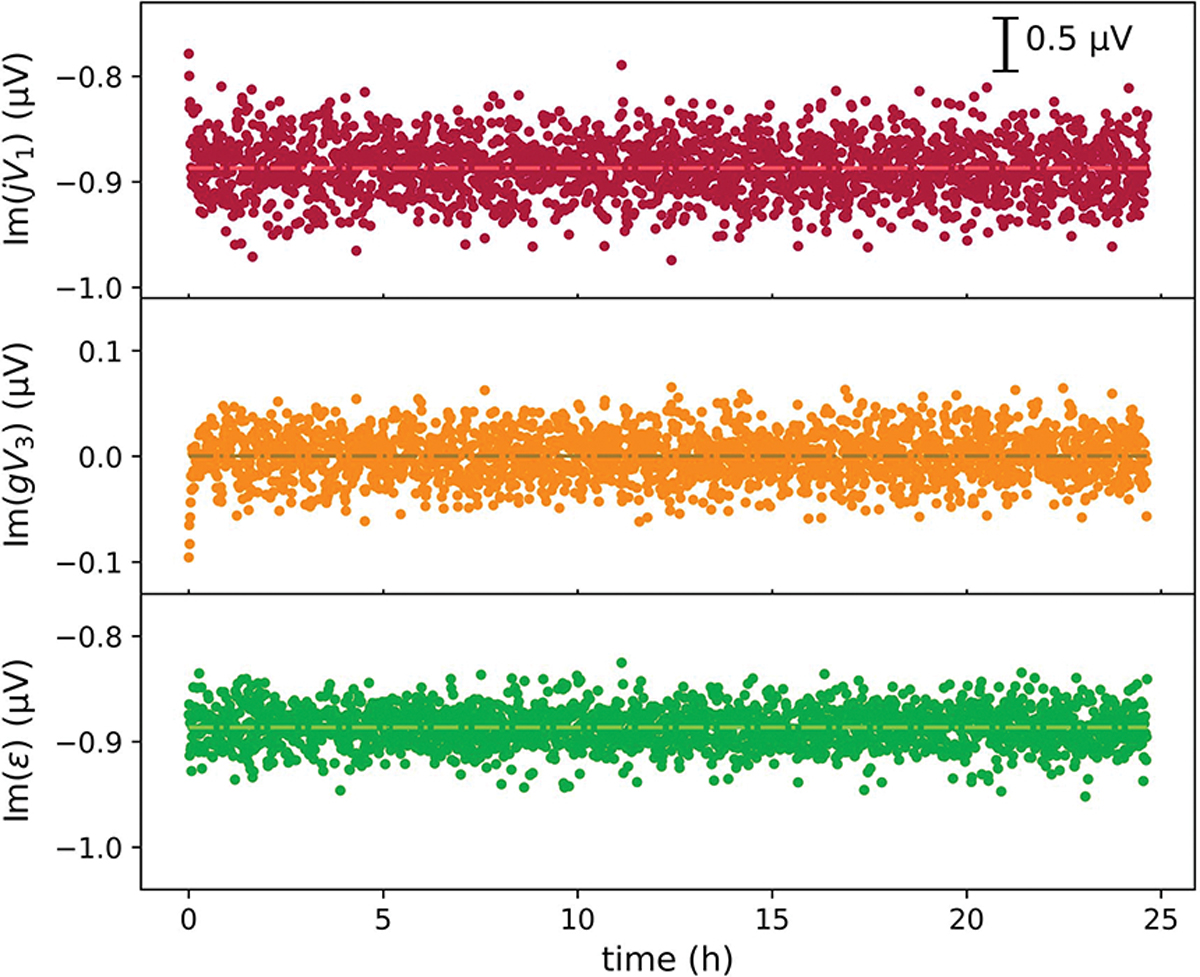
Imaginary components of recorded voltages as function of time: 1) $jV_{1}$; 2) $V_{3}$ scaled with the gain factor; and (3) ε.

A major advantage of the digital bridge is that the excitation voltages and the error signal can be fully digitized, and the bridge dynamics can be analyzed in postprocessing. All the test results presented herein were acquired with the bridge setup shown in Fig. 2. The gain of the transimpedance amplifier was set at 10^7^ V/A, and the corresponding $Z_{3}$ was approximately 10 M; the 3-dB bandwidth at this setting is 50 kHz. Figs. 3 and 4 show the measured $V_{1}$, $V_{2}$, $V_{3}$ values, and the bridge error voltage, ε, as a function of time that were acquired with $S_{1}$ and $S_{2}$ set at 1233.19734 Hz, a phase of 90° and 0°, and an amplitude of 10 and 0.1 V, respectively. The complex amplitude of $S_{3}$ set at the same frequency was automatically controlled through a computer to minimize the mean bridge error $\left(V_{3}\right)$, using a simple proportional-integral feedback algorithm.

The phase difference between $V_{1}$ and $V_{2}$ is approximately −90°. The digitized voltages are phase normalized such that the phase of $V_{2}$ is 0. Their complex components are more conveniently compared between $jV_{1}$ and $V_{2}$. The real parts of $jV_{1}$ and $V_{2}$ (see Fig. 3) fluctuated, on the order of 10 μV, exceeding a factor of 10 more than the imaginary counterparts (see Fig. 4). This reflects that the digital sources have better phase stabilities than amplitude stabilities. The real component of $V_{2}$ closely follows that of $jV_{1}$, resulting from the feedback action that minimizes the bridge error signal.

For better comparison, the error voltage $V_{3}$ is shown after being scaled with an estimated gain factor. Re(gV3) is dominated by white noise, and its mean is effectively locked to 0 through the feedback (see Fig. 3). The fluctuations of Im(gV3) form a mirror image of Im(jV1), with its mean also locked to 0 (see Fig. 4).

We can qualitatively understand how the detected error voltage $V_{3}$ relates to the source fluctuation δV by considering that the transimpedance amplifier together with $Z_{1}$ and $Z_{2}$ form a summing amplifier. Since $Z_{1}/k_{o}$ and $Z_{2}$ are nominally equal in magnitude and differ by 90° in phase, we have $V_{1}+jV_{2}\approx -\delta V$.

We define

(3) $$\varepsilon =j\delta V+gV_{3}.$$

The real and imaginary components of ε are shown in Figs. 3 and 4, respectively. Both components follow a white noise distribution, with a standard deviation of less than 0.05 μV, indicating a strong correlation between δV and $gV_{3}$.

### C. Correlation Analysis and Noise Cancellation

To analyze the dynamics of the bridge balancing more rigorously, we applied Kirchhoff’s law to the bridge circuit

(4) $$\frac{k_{o}V_{1}}{Z_{1}}+\frac{V_{2}}{Z_{2}}+\frac{V_{3}}{Z_{3}}=0.$$

Using conventional notations, we define α and β as the real and imaginary part of the deviation, respectively, from the nominal impedance ratio that is perfectly matched to the IVD ratio in magnitude

(5) $$\frac{Z_{1}}{Z_{2}}=\lambda (1+\alpha +j\beta )$$

where $\lambda =jk_{o}$.

Combining (4) and (5), we rewrite

(6) $$1-\frac{jV_{1}}{V_{2}}=-\alpha -j\beta -\frac{1}{\lambda }\frac{Z_{1}}{Z_{3}}\frac{V_{3}}{V_{2}}.$$

We define

(7) $$u=1-\frac{jV_{1}}{V_{2}}$$

(8) $$v=\frac{V_{3}}{V_{2}}.$$

Equation (6) becomes

(9) u=−α−jβ+gv.

Using a linear fitting between the complex variables u and v, we can determine g. We then have

(10) α=Re(gv−u)

(11) β=Im(gv−u).

**Fig. 5.**
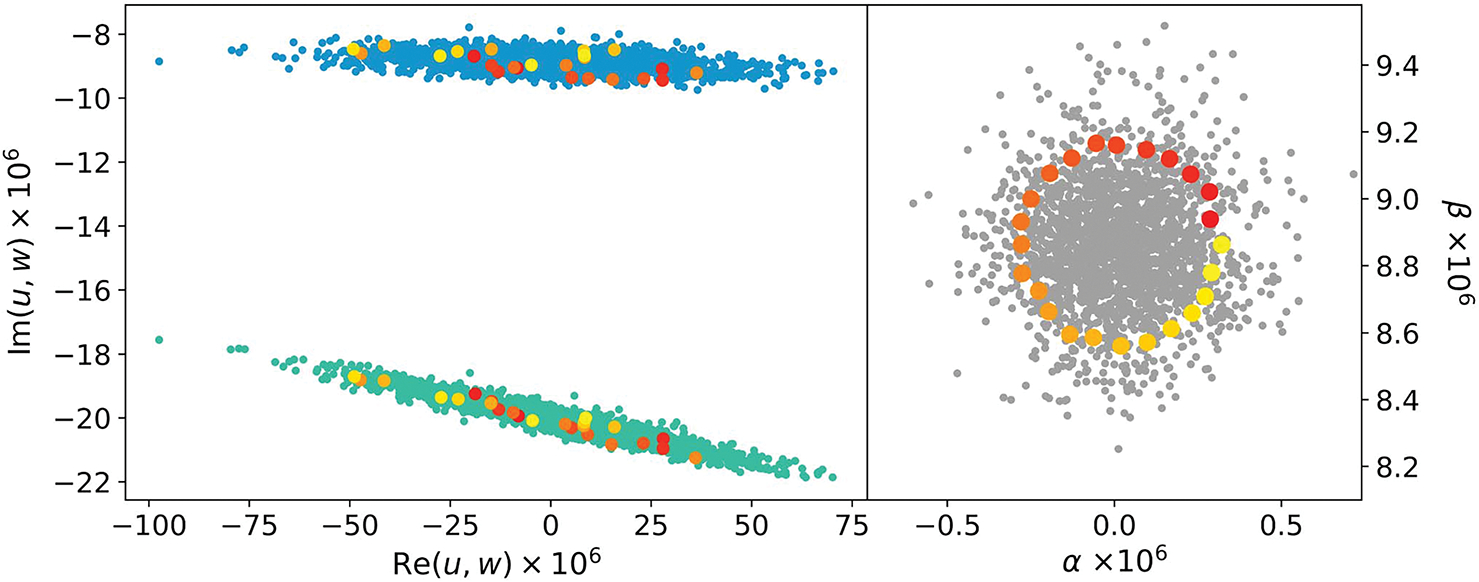
(a) Imaginary part versus real part: u in light blue and w in cyan. w shifted lower by j 20 × 10^−6^ for clarity. (b) β versus α. 24 data points of the fitting residuals distributed close to the perimeter of a circle are colored progressively in (b); the corresponding u and w points in (a) show their correlation.

To visualize the effectiveness of the linear fitting, we plot the imaginary part versus the real part for u and w=|g|v in Fig. 5(a). The natural fluctuation of u is mainly along the real axis, covering a range of about 150 μV/V, reflecting that the digital sources have better phase stabilities than amplitude stabilities. The pattern of w is similar to that of u, except that it is tilted due to a phase shift of the current amplifier. The residuals of the linear fitting can be seen in Fig. 5(b), showing α versus β. The residual data points distribute tightly in a circle of radius about 0.5 μV/V, indicating that the fluctuations of u and v largely cancel out in determining α and β.

**Fig. 6.**
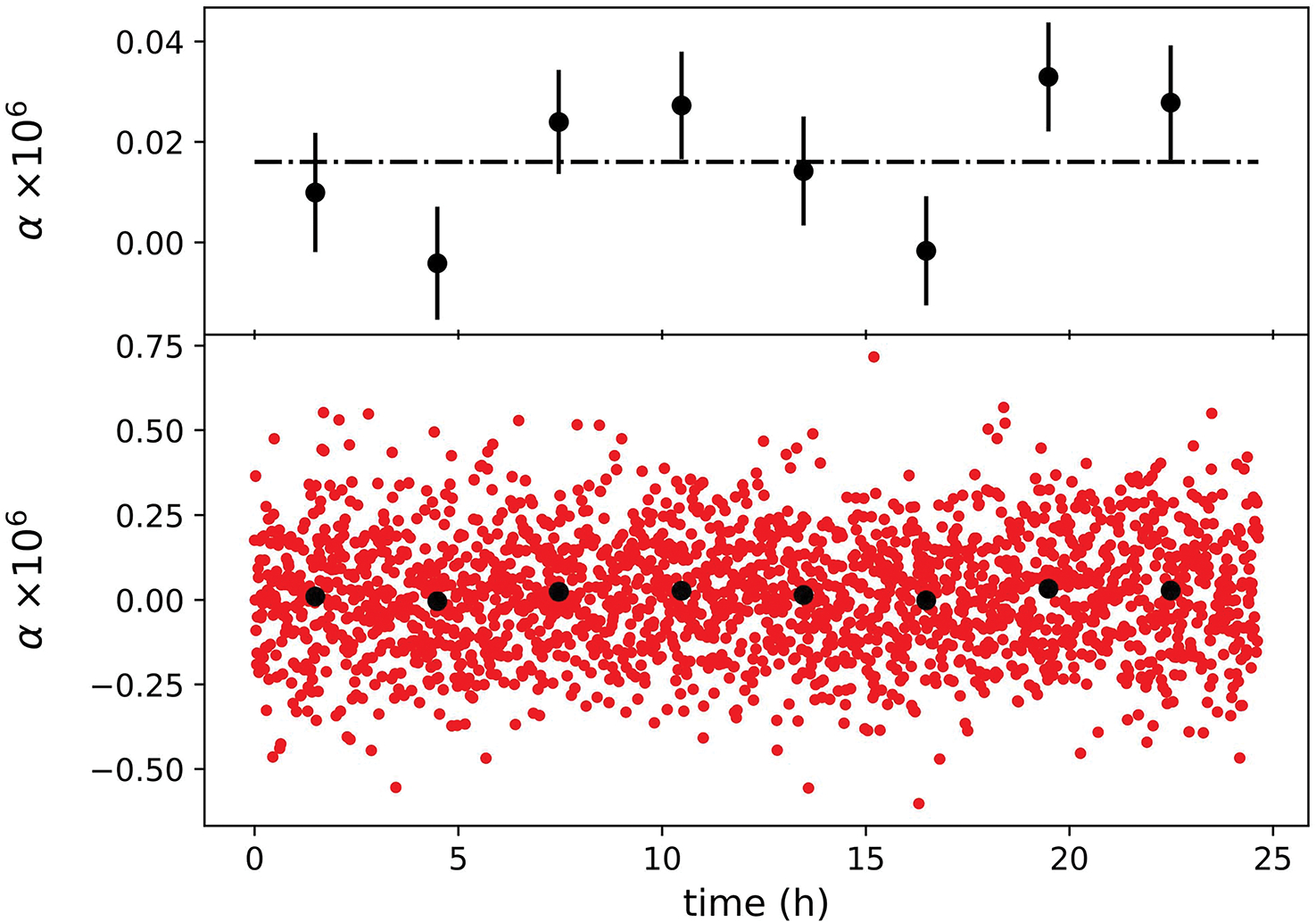
Determined α as a function of time. The black dots were obtained by averaging 256 points, or about 2 h worth of data. The error bars in the top graph denote the 1-σ standard deviation of the 256 points.

Fig. 6 shows α as a function of time over a period of 24 h. The distribution of the data points is consistent with a constant that is buried in white noise. Each data point in the lower panel takes about 36 s to acquire, and all the data points stay within ±0.7 × 10^−6^. Averaging 256 points, or about 2 h worth of data, produces a new set of averaged data that fluctuates within ±0.02 × 10^−6^ about their mean. The fluctuations can be attributed predominantly to the limited resolution of the digitizer.

**Fig. 7.**
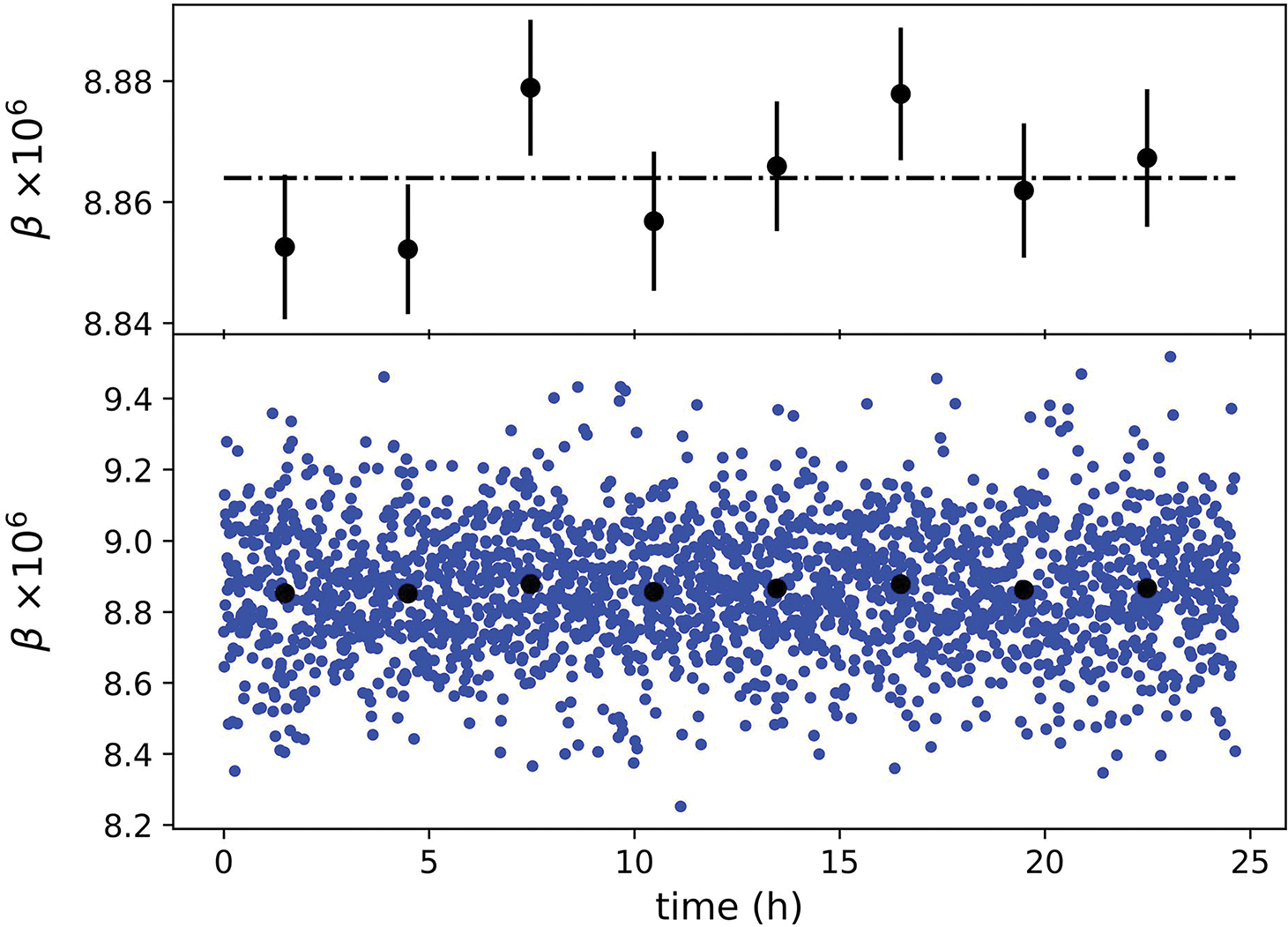
Determined β as a function of time. The black dots were obtained by averaging 256 points, or about 2 h worth of data. The error bars in top graph denote the 1-σ standard deviation of the 256 points.

Fig. 7 shows β as a function of time over the same period. The distribution of the data points of β are similar to α and also consistent with a constant value over time. All the data points stay within ±0.7 × 10^−6^. Averaging 256 points also produces a new set of averaged data that fluctuates within ±0.02 × 10^−6^ about their mean.

**Fig. 8.**
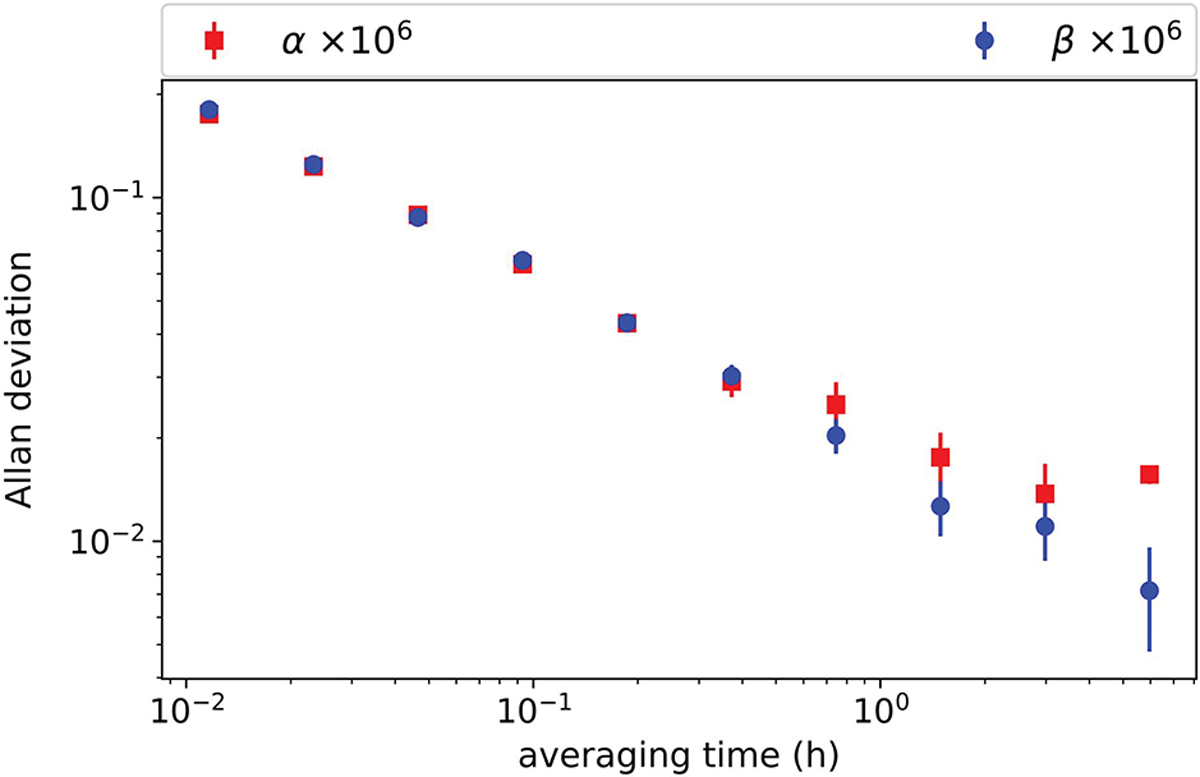
Squares and circles are the real and imaginary parts of the deviation from the nominal impedance ratio. Error bars are 1-σ standard deviation of the Allan deviation.

The Allan deviations of α and β are shown in Fig. 8. Both decrease to 2 × 10^−8^ level in about 3 h and show a monotonic downward trend over the test time window, demonstrating the stability of the digital bridge.

### D. Results and Uncertainty Analysis

The digital impedance bridge enables us to measure the capacitance of C in reference to $R_{H}$ with a Type A uncertainty (k=1) of 0.02 μF/F. Repeated measurements show that the results of C using the digital bridge are consistent, within 0.11 μF/F, with its capacitance measured against the Farad Bank, which is used to maintain the capacitance unit at the National Institute of Standards and Technology (NIST), Gaithersburg, MD, USA, and is traceable to the calculable capacitor [17]. The difference can be partly attributed to the frequency dependence of C because the digital bridge functions near 1233 Hz to keep the impedance ratio close to 100:1 in magnitude while the capacitance measurement relative to the Farad Bank has been restricted to 1592 Hz. However, the largest uncertainty source for the digital bridge is the digitizer error as shown in Table I.

The digital errors associated with the digitizer may arise from aliasing and spectral leakage. Stray capacitances in the digitizer may also cause crosstalk between the ADC channels and leakage to the ground. These errors have been estimated experimentally and numerically by varying the sampling rate and the record length, combined with temporarily introducing extra cross capacitances and changing from the differential input mode to the single-ended mode. Sensitivity to harmonics has been estimated experimentally and numerically by including selected harmonic base functions in the sine fit, adding simulated harmonic content to the digitized data record before the sine fit, and physically injecting additional third harmonic voltage into the bridge excitation. Possible offset error in the detected $V_{3}$ due to non-linearity of the current amplifier and the ADC, causing intermodulation distortion, was also accessed by changing the gain settings of the amplifier and the ADC; no correlated change was detected within the limit of the bridge resolution.

In the future, we plan to modify the front analog circuit of the digitizer to reduce its error. The uncertainty for the frequency dependence determination, which has currently been limited by the stability of a reference 1-pF cross capacitor at NIST, can also be significantly reduced [17], [18].

## IV. CONCLUSION

We evaluated a digital impedance bridge in a hybrid structure for comparison of a capacitor with a resistor where the impedance ratio was measured in two separate parts. The modulus of the impedance ratio was matched arbitrarily close to the input-to-output ratio, in magnitude, of a two-stage IVD by adjusting the operating frequency of the bridge; the residual deviation between the two together with the phase factor of the impedance ratio was measured using a custom detection system based on a four-channel 24-bit digitizer. The IVD was calibrated, *in situ*, using a four-arm bridge with two known capacitors. In contrast to the conventional approach of emphasizing precision and stability of the voltage sources driving the bridge, we adopted an approach that focused on the resolution and stability of the detectors. Fluctuations of the source voltages were largely removed through postprocessing of the digitized data, and the measurement results were limited by the digitizer error. While we have achieved a low Type A uncertainty (k=1) of 0.02 μF/F in 2 h for determining the capacitance of a 100-pF capacitor relative to a 12 906-Ω resistor at 1233 Hz, the combined relative standard uncertainty (k=1) is 0.13 μF/F. Even though the uncertainty is not as low as for a conventional IVD-based double-quadrature bridge which has the modulus of the nominal impedance ratio equal to one, the digital bridge discussed here has a key advantage. The modulus of the nominal impedance ratio of the digital bridge is 100. This approach has the advantage of shortening the measurement chain from a 12 906-Ω resistor to a 100-pF capacitor by two 10:1 ratio steps. In the future, we plan to focus our research on reducing the digitizer error for the digital impedance bridge to serve as an alternative system at NIST for realizing the capacitance unit.

The detection system based on the DAQM909A for measuring ac voltage ratios compares favorably to the system based on the SR860 lock-in detectors which we evaluated previously [15]. We achieved a factor of 10 improvement in terms of the Allan deviations for the impedance ratio measurements over a comparable averaging window. We can attribute the improvement to the higher resolution of the modern data acquisition board and the customized demodulation method in post-processing, which is not accessible with the commercial lock-in detectors.

## Figures and Tables

**TABLE I:** Uncertainty Budget (*k* = 1)

	Relative standard uncertainty (×10^−6^)
Type A	0.02
Digitizer error	0.10
Frequency dependence of *C_a_* (1 pF) and *C_b_* (100 pF)	0.07
*C_a_* and *C_b_* relative to Farad Bank	0.03
*R_H_* relative to dc QHR	0.01
Frequency dependevce of *R_H_*	0.01
Relative combined standard uncertainty	0.13

## References

[1] Overney F and Jeanneret B, “Impedance bridges: From Wheatstone to Josephson,” Metrologia, vol. 55, no. 5, pp. S119–S134, Jul. 2018.

[2] Thompson AM, “An absolute determination of resistance based on a calculable standard of capacitance,” Metrologia, vol. 4, no. 1, pp. 1–7, 1968.

[3] Cutkosky RD, “Techniques for comparing four-terminal-pair admittance standards,” J. Res. Nat. Bur. Standards, vol. 74C, nos. 3–4, p. 63, Jul. 1970.

[4] Schurr J, Bürkel V, and Kibble BP, “Realizing the Farad from two AC quantum Hall resistances,” Metrologia, vol. 46, no. 6, pp. 619–628, 2009.

[5] Overney F , “Josephson-based full digital bridge for high-accuracy impedance comparisons,” Metrologia, vol. 53, no. 4, pp. 1045–1053, 2016. 10.1088/0026-1394/53/4/1045 PMC13464339 42592508

[6] Bauer S , “A four-terminal-pair Josephson impedance bridge combined with a graphene-quantized Hall resistance,” Meas. Sci. Technol, vol. 32, no. 6, Mar. 2021, Art. no. 065007.

[7] Ramm G and Moser H, “New multifrequency method for the determination of the dissipation factor of capacitors and of the time constant of resistors,” IEEE Trans. Instrum. Meas, vol. 54, no. 2, pp. 521–524, Apr. 2005.

[8] Kucera J, Funck T, and Melcher J, “Automated capacitance bridge for calibration of capacitors with nominal value from 10 nF up to 10 mF,” in Proc. Conf. Precis. Electromagn. Meas., Jul. 2012, pp. 596–597.

[9] Rybski R, Kaczmarek J, and Kontorski K, “Impedance comparison using unbalanced bridge with digital sine wave voltage sources,” IEEE Trans. Instrum. Meas, vol. 64, no. 12, pp. 3380–3386, Dec. 2015.

[10] Kučera J, Kováč J, Palafox L, Behr R, and Vojáčková L, “Characterization of a precision modular sinewave generator,” Meas. Sci. Technol, vol. 31, no. 6, Jun. 2020, Art. no. 064002.

[11] Marzano M, Ortolano M, D’Elia V, Müller A, and Callegaro L, “A fully digital bridge towards the realization of the Farad from the quantum Hall effect,” Metrologia, vol. 58, no. 1, Feb. 2021, Art. no. 015002.

[12] Ihlenfeld WGK and Vasconcellos RTB, “A digital four terminal-pair impedance bridge,” in Proc. Conf. Precis. Electromagn. Meas. (CPEM), Jul. 2016, pp. 1–2.

[13] Homan DN, “Applications of coaxial chokes to A-C bridge circuits,” J. Res. Nat. Bur. Standards, vol. 72C, no. 2, pp. 161–165, 1968.

[14] Small GW, Fiander JR, and Coogan PC, “A bridge for the comparison of resistance with capacitance at frequencies from 200 Hz to 2 kHz,” Metrologia, vol. 38, no. 4, pp. 363–368, 2001.

[15] Feige M, Schlamminger S, Waltrip B, Berilla M, and Wang Y, “Evaluations of a detector-limited digital impedance bridge,” J. Res. Nat. Inst. Standards Technol, vol. 126, Apr. 2021, Art. no. 126006. 10.6028/jres.126.006 PMC9681207 36475083

[16] IEEE Standard for Digitizing Waveform Recorders, IEEE Standard 1057–2017, Jan. 2018.

[17] Gournay P , “Comparison CCEM-K4.2017 of 10 pF and 100 pF capacitance standards,” Metrologia, vol. 56, no. 1A, p. 01001, 2019.

[18] Wang Y, Shields S, “Improved capacitance measurements with respect to a 1-pF cross-capacitor from 200 to 2000 Hz,” IEEE Trans. Instrum. Meas, vol. 54, no. 2, pp. 542–545, Apr. 2005.

